# Association Between the Composite Cardiovascular Risk and mHealth Use Among Adults in the 2017-2020 Health Information National Trends Survey: Cross-Sectional Study

**DOI:** 10.2196/46277

**Published:** 2024-01-04

**Authors:** Yuling Chen, Ruth-Alma Turkson-Ocran, Binu Koirala, Patricia M Davidson, Yvonne Commodore-Mensah, Cheryl Dennison Himmelfarb

**Affiliations:** 1 Johns Hopkins University School of Nursing Baltimore, MD United States; 2 Beth Israel Deaconess Medical Center/Harvard Medical School Boston, MA United States; 3 University of Wollongong New South Wales Australia; 4 Johns Hopkins University Bloomberg School of Public Health Baltimore, MD United States; 5 Johns Hopkins University School of Medicine Baltimore, MD United States

**Keywords:** mobile health, usage, cardiovascular risk, association, mobile phone

## Abstract

**Background:**

Numerous studies have suggested that the relationship between cardiovascular disease (CVD) risk and the usage of mobile health (mHealth) technology may vary depending on the total number of CVD risk factors present. However, whether higher CVD risk is associated with a greater likelihood of engaging in specific mHealth use among US adults is currently unknown.

**Objective:**

We aim to assess the associations between the composite CVD risk and each component of mHealth use among US adults regardless of whether they have a history of CVD or not.

**Methods:**

This study used cross-sectional data from the 2017 to 2020 Health Information National Trends Survey. The exposure was CVD risk (diabetes, hypertension, smoking, physical inactivity, and overweight or obesity). We defined low, moderate, and high CVD risk as having 0-1, 2-3, and 4-5 CVD risk factors, respectively. The outcome variables of interest were each component of mHealth use, including using mHealth to make health decisions, track health progress, share health information, and discuss health decisions with health providers. We used multivariable logistic regression models to examine the association between CVD risk and mHealth use adjusted for demographic factors.

**Results:**

We included 10,531 adults, with a mean age of 54 (SD 16.2) years. Among the included participants, 50.2% were men, 65.4% were non-Hispanic White, 41.9% used mHealth to make health decisions, 50.8% used mHealth to track health progress toward a health-related goal, 18.3% used mHealth to share health information with health providers, and 37.7% used mHealth to discuss health decisions with health providers (all are weighted percentages). Adults with moderate CVD risk were more likely to use mHealth to share health information with health providers (adjusted odds ratio 1.49, 95% CI 1.24-1.80) and discuss health decisions with health providers (1.22, 95% CI 1.04-1.44) compared to those with low CVD risk. Similarly, having high CVD risk was associated with higher odds of using mHealth to share health information with health providers (2.61, 95% CI 1.93-3.54) and discuss health decisions with health providers (1.56, 95% CI 1.17-2.10) compared to those with low CVD risk. Upon stratifying by age and gender, we observed age and gender disparities in the relationship between CVD risk and the usage of mHealth to discuss health decisions with health providers.

**Conclusions:**

Adults with a greater number of CVD risk factors were more likely to use mHealth to share health information with health providers and discuss health decisions with health providers. These findings suggest a promising avenue for enhancing health care communication and advancing both primary and secondary prevention efforts related to managing CVD risk factors through the effective usage of mHealth technology.

## Introduction

Cardiovascular disease (CVD) remains the leading cause of mortality in the United States and worldwide [1]. CVD-related mortality can be reduced by controlling modifiable major CVD risk factors, including diabetes mellitus, hypertension, physical inactivity, smoking, and overweight or obesity [2]. Despite the development and implementation of interventions to manage CVD risk factors [3-9], the high prevalence of CVD risk factors persists among both US adults with and without a history of CVD [10].

Mobile health (mHealth) technology is becoming an increasingly important part of the self-management of CVD risk factors facilitated by the widespread adoption of smartphones and tablets [11]. In 2018, a total of 73% of US adults with or at risk for CVD owned a smartphone or tablet [12]. Evidence showed that mHealth technology is beneficial for both primary and secondary prevention of CVD [13,14]. Some systematic reviews, meta-analyses, and clinical trials indicate that mHealth interventions may promote physician-patient communications, control modifiable CVD risk factors, and support cardiovascular health, and the effectiveness of mHealth intervention varied by the frequency of mHealth use [12,15-22].

Several studies have examined disparities in mHealth use among individuals with and without CVD risk factors, which indicated that the connection between CVD risk and mHealth use might exhibit variations depending on the total number of CVD risk factors present. For example, a nationally representative cross-sectional study of 3248 US adults from 2018 Health Information National Trends Survey (HINTS) found that individuals diagnosed with CVD or having CVD risk factors (including diabetes mellitus, hypertension, and current smoking) were more likely to use mHealth to share health information compared with those without CVD or the above CVD risk factors [12]. Nonetheless, it is significant to acknowledge that the study [12] omitted specific CVD risk factors, including physical activity and obesity, which could substantially influence mHealth use [23]. Additionally, the extent to which mHealth use differs based on the combination of CVD risk factors (including diabetes mellitus, hypertension, current smoking, physical inactivity, and obesity) was not explored in that study [12]. Another large cross-sectional study of 256,117 US adults from 2011 to 2018 National Health Interview Survey indicated that health information technologies use among adults with a history of CVD was lower than that of health information technologies use among adults without a history of CVD [15]. Similarly, a cross-sectional study of 28,948 US adults from 2012 to 2018 National Health Interview Survey found that health information technologies use was highest among adults with no CVD risk factors, followed by adults with 1 risk factor, and was lowest among adults with multiple CVD risk factors (including diabetes, hypertension, and obesity) [16]. Of note, these 2 studies mainly focus on the use of health information technologies, such as looking up health information on the internet, filling a web-based prescription, scheduling a medical appointment on the internet, communicating with health care providers through email, or using web-based group chats to learn about health topics [15,16]. Whether higher CVD risk is associated with a greater likelihood of engaging in specific mHealth use (such as sharing health information with health providers) among US adults, regardless of whether they have a history of CVD or not while controlling for demographic variables, is currently unknown.

Moreover, past studies have shown age and gender disparities in mHealth use, where older adults and women were less likely to use mHealth than their younger and male comparators, respectively [10,15,17,19,24-26]. However, how the associations between CVD risk and mHealth use differed by age and gender is not fully elucidated. Thus, we sought to assess the associations between the composite CVD risk and mHealth use among US adults using national data. We also aimed to compare the relationship of the composite CVD risk with mHealth use among younger (<65 years) and older adults (≥65 years) and between women and men to better understand the complex relationship between CVD risk and mHealth use.

## Methods

### Study Design and Setting

The HINTS is a nationally representative cross-sectional study that has been administered every few years by the National Cancer Institute. The HINTS target population is civilian, noninstitutionalized adults aged 18 years or older living in the United States. HINTS has been administered every few years since 2003, including HINTS 1 (2003), HINTS 2 (2005), HINTS 3 (2008), HINTS 4 (Cycle 1-4, 2011-2014), and HINTS 5 (Cycle 1-4, 2017-2020). Our analysis used HINTS 5 Cycle 1 (2017), HINTS 5 Cycle 2 (2018), HINTS 5 Cycle 3 (2019), and HINTS 5 Cycle 4 (2020) data sets. Cycles 1, 2, and 3 data were collected from January through May in 2017, 2018, and 2019; Cycle 4 data were collected from February through June 2020. The survey (HINTS 5 Cycle 1-4) was conducted exclusively by mail with a US $2 prepaid monetary incentive to encourage participation. In Cycles 1, 2, and 4, the survey was conducted using a paper-based format. In Cycle 3, the survey encompassed both a paper-based version and a web-based survey (mailed contact materials containing a link to the web-based survey). Detailed mailing protocol and full description of the HINTS methodologies can be found elsewhere [27,28]. To increase the precision of estimates for minority subpopulations, the sampling frame of addresses was grouped into high- and low-minority strata and included oversampling of the high-minority stratums. We pooled the data sets from the HINTS 5 Cycle 1-4 to increase the precision of the estimates [27]. Our study adheres to the reporting guidelines outlined in the STROBE (Strengthening the Reporting of Observational Studies in Epidemiology).

### Study Population

The target population of this analysis comprises adults aged 18 years and older regardless of whether they have a history of CVD or not (n=16,092). That is, both adults with and without CVD were included in our analysis. CVD status was determined based on the question: “Has a doctor or other health professional ever informed you that you had a heart condition such as heart attack, angina, or congestive heart failure?”

**Figure 1:**
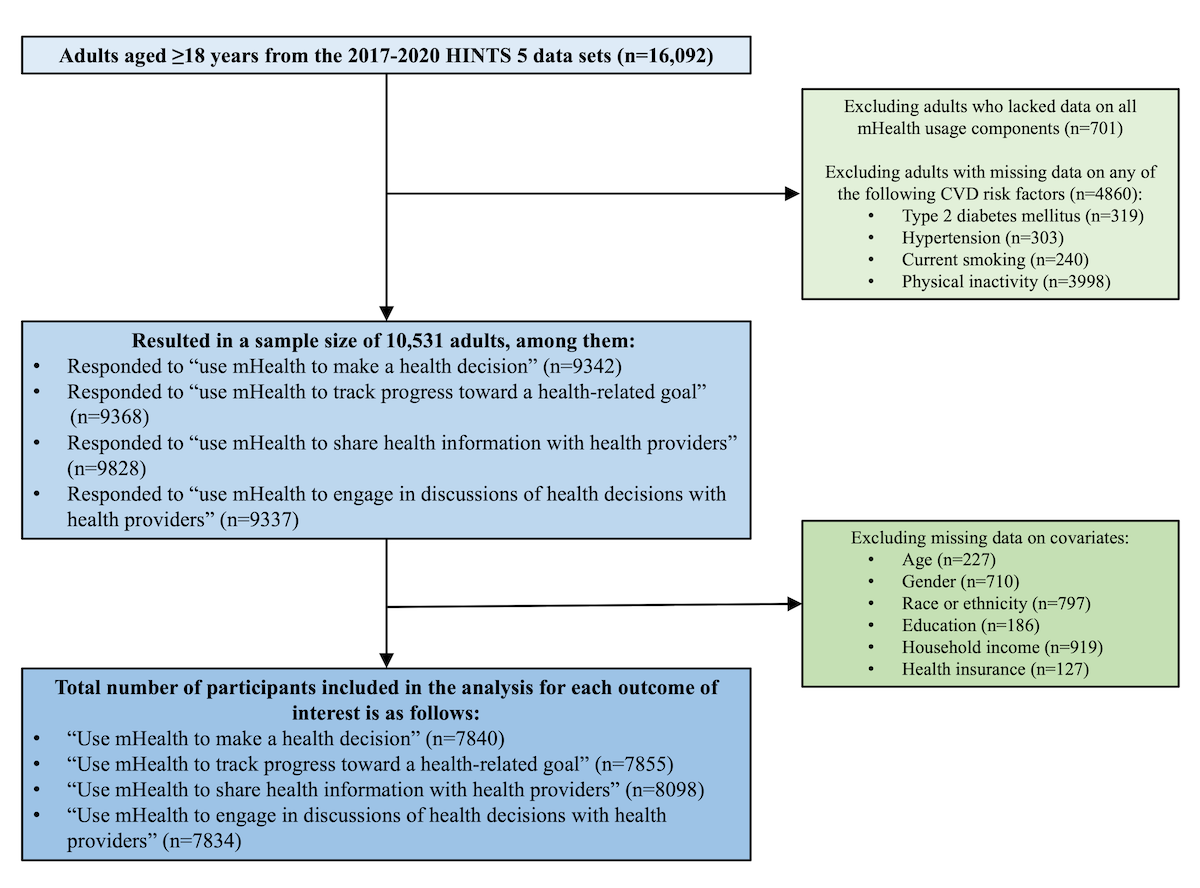
Flowchart of participant selection process. CVD: cardiovascular disease; HINTS: Health Information National Trends Survey; mHealth: mobile health.

Figure 1 illustrates the flowchart outlining the participant selection process. As the outcomes of interest are each component of mHealth use, we initially excluding those adults who lacked data on all mHealth use components (see definition in the *mHealth Use Assessment* section below). Then, we excluded adults with missing data on any of the CVD risk factors (including type 2 diabetes mellitus, hypertension, current smoking, physical inactivity, and overweight or obesity; see definition in the *Cardiovascular Risk Measurements* section below) to ensure the variable of exposure, the composite CVD risk, is complete. By excluding adults lacking data on all mHealth use components (n=701) and those with missing data on any of the CVD risk factors (n=4860), this study’s sample comprised 10,531 adults, including 806 with CVD and 9725 without CVD (refer to Table S1 in Multimedia Appendix 1 for details). Among the 10,531 participants, 9342 responded to the use of mHealth to make a health decision, 9368 responded to the use of mHealth to track progress toward a health-related goal, 9828 responded to the use of mHealth to share health information with health providers, and 9337 responded to the use of mHealth to engage in discussions of health decisions with health providers (Table S2 in Multimedia Appendix 1).

After excluding adults with missing data on covariates (see definition in the *Covariates* section below), the total number of participants included in the analysis for each outcome of interest was as follows: 7840 participants for the outcome of using mHealth to make a health decision, 7855 participants for the outcome of using mHealth to track progress toward a health-related goal, 8098 participants for the outcome of using mHealth to share health information with health providers, and 7834 participants for the outcome of using mHealth to engage in discussions of health decisions with health providers.

### Ethical Considerations

HINTS has received approval from the Westat Institutional Review Board and has been designated as exempt by the US National Institutes of Health Office of Human Subjects Research Protections due to the deidentification of the data. Analyses using the HINTS database met the criteria for research involving nonhuman subjects, as determined by the Johns Hopkins University School of Medicine Institutional Review Board, and this analysis did not require review. An expedited approval of HINTS was obtained for project number 6048.14 (FWA 00005551)

### mHealth Use Assessment

The outcome variables of interest consist of each component of mHealth use, which was measured by four self-reported “yes/no” questions as follows: (1) “Has your tablet or smartphone helped you track progress toward a health-related goal, such as quitting smoking, losing weight, or increasing physical activity?” (2) “Has your tablet or smartphone helped you make a decision about how to treat an illness or condition?” (3) “Have you shared health information from either an electronic monitoring device or smartphone with a healthcare professional within the last 12 months?” and (4) “Has your tablet or smartphone helped you in discussions of health decisions with your health providers?”

### Cardiovascular Risk Measurements

The exposure of interest in this study was CVD risk, defined as the presence of CVD risk factors. The CVD risk factors assessed in this analysis included type 2 diabetes mellitus, hypertension, current smoking, physical inactivity, and overweight or obesity. Each CVD risk factor was recorded as “0” or “1.” According to a previous study [29], we defined low, moderate, and high CVD risk as having 0-1, 2-3, and 4-5 CVD risk factors, respectively.

Diabetes mellitus and hypertension were assessed by 2 self-reported questions: “Has a doctor or other healthcare professional ever told you that you had diabetes or high blood sugar?” and “Has a doctor or other healthcare professional ever told you that you had high blood pressure or hypertension?” Having diabetes or hypertension was recorded as “1.”

Smoking status was classified as “current smoking” or “current non-smoking” at the time of the survey. Current smoking was defined as the current use of cigarettes, determined through the combination of the following 2 questions: “Have you smoked at least 100 cigarettes in your entire life?” and “How often do you now smoke cigarettes? (Responses: every day, some days, or not at all).” Current smoking was recorded as “1.”

Physical inactivity was defined as <150 minutes of at least moderate-intensity activity each week. Physical activity was derived from the combination of two questions: (1) “How many days do you do any at least moderate physical activity or exercise? (Responses: none, 1 day per week, 2 days per week, 3 days per week, 4 days per week, 5 days per week, 6 days per week, and 7 days per week)” and (2) “How long are you typically doing these activities (minutes and hours)?” Having physical inactivity was recorded as “1.”

BMI was initially divided into 6 categories according to World Health Organization (WHO) recommendations: 15.0-18.5 kg/m^2^ (underweight), 18.5-24.9 kg/m^2^ (normal weight), 25.0-29.9 kg/m^2^ (overweight), 30.0-34.9 kg/m^2^ (obesity level I), 35.0-39.9 kg/m^2^ (obesity level II), and 40-59.9 kg/m^2^ (obesity level III) [29]. Having overweight or obesity (level I, II, or III) was recorded as “1.”

### Covariates

Other covariates examined included baseline age, gender, race, educational levels, household income, location, and health insurance [12]. Age at the time of the interview was divided into 5 categories as follows: 18-34, 35-49, 50-64, 65-74, and 75 years or older. Race was categorized as Hispanic, non-Hispanic Asian, non-Hispanic Black or African American, non-Hispanic others, and non-Hispanic White. Educational levels were classified as less than high school, 12 years or completed high school, some college, and college graduate or higher. Household income was categorized as less than US $20,000; US $20,000-$35,000; US $35,000-$50,000; US $50,000-$75,000; and US $75,000 or more. HINTS comprises 8 questions that inquire about the different types of health insurance held by participants, including insurance obtained through a current or former employer or union, insurance purchased directly from an insurance company, and similar categories. The responses to these questions were compiled into a derived measure of whether or not any health insurance covered the respondent. As a result, health insurance was categorized into 2 groups: “yes” or “no.” The location (whether rural or urban) was determined using a single variable available in the HINTS data set, which was classified into categories including metropolitan: large metro, metropolitan: large fringe metro, metropolitan: medium metro, metropolitan: small metro, nonmetropolitan: micropolitan, and nonmetropolitan: noncore. These categories were based on the 2013 National Center for Health Statistics urban-rural classification scheme for counties. We recategorized this variable into 2 groups: rural (nonmetropolitan) and urban (metropolitan) for our analysis.

### Statistical Analysis

We described the demographic characteristics and mHealth use using both unweighted and weighted percentages by CVD risk (low, moderate, and high CVD risk). Based on the analytic methods suggested by the *HINTS Users Data Handbook*, we used the survey weighting and Taylor series variance estimation to calculate the prevalence estimated and SEs [27]. Using survey-weighted Pearson chi-square tests by performing “svy:tabulate” command in Stata/SE (version: 17.0; Stata Corp LLC) software, we compared the demographic variables and each component of mHealth use by CVD risk [27,30,31]. We initially ran a full multivariable logistic regression model with survey-weighting to examine the association between the CVD risk category and each component of mHealth use, adjusting for age, gender, race, educational levels, household income, location, and health insurance. Then, we conducted multivariable logistic regression models stratifying by age category (<65 and ≥65 years) and gender.

We also conducted sensitivity analyses excluding individuals previously diagnosed with CVD and individuals aged ≥85 years. In addition, to explore potential variations in the associations between CVD risk factors and mHealth use, we performed 4 distinct weighted multivariate logistic regression models, with each component of mHealth use serving as the dependent variable. In each model, the independent variables included the 5 CVD risk factors (type 2 diabetes mellitus, hypertension, current smoking, physical inactivity, and overweight or obesity), and was adjusted for the previously mentioned covariates.

We performed all statistical analyses using the “svy” command in Stata/SE (version 17.0). In multivariable logistic regression analyses, we excluded cases with missing data on any of the outcome variables or covariates as the default approach. Adjusted odds ratio and 95% CI were calculated for multivariable logistic regression models. A 2-sided *P* value of <.05 was considered statistically significant for all analyses.

## Results

We included 10,531 adults, with a mean age of 54 (SD 16.2) years (Table S2 in Multimedia Appendix 1). Table 1 displays the survey-weighted demographic characteristics of the included participants. Of these, 50.2% (weighted percentage) were men, 65.4% (weighted percentage) were non-Hispanic White, 36.9% (weighted percentage) had a bachelor’s degree, and 45.3% (weighted percentage) had an annual income US $75,000. Unweighted participants’ proportions of demographic characteristics by CVD risk can be found in Table S2 in Multimedia Appendix 1.

**Table 1:** Weighted demographic characteristics by cardiovascular risk among adults with or at risk for CVD^a^ (N=10,531).

Characteristics			All (N=10,531; weighted percentage, %)		Low CVD risk^b^ (n=4332; weighted percentage, %)		Moderate CVD risk^c^ (n=5316; weighted percentage, %)		High CVD risk^d^ (n=883; weighted percentage, %)		*P* value	
**Age group (y)**												<.001
	18-34	26.91		37.74		19.89		3.45				
	35-49	27.78		27.31		28.88		23.25				
	50-64	29.76		23.82		33.10		46.18				
	65-74	10.57		7.57		12.32		18.47				
	≥75	4.98		3.56		5.81		8.65				
**Gender**												.006
	Women	49.80		52.83		47.06		48.59				
	Men	50.20		47.17		52.94		51.41				
**Race and ethnicity**												<.001
	Hispanic	15.93		16.27		15.37		17.60				
	Non-Hispanic Asian	5.55		6.49		4.90		3.69				
	Non-Hispanic Black	10.02		7.07		11.87		17.20				
	Non-Hispanic others	3.08		3.10		3.18		2.22				
	Non-Hispanic White	65.42		67.07		64.68		59.29				
**Education**												<.001
	Less than high school	5.68		3.24		6.91		13.40				
	High school graduate	18.91		15.45		20.83		28.68				
	Some college	38.50		36.61		40.15		39.71				
	Bachelor's degree	36.91		44.70		32.11		18.21				
**Household income (US $)**												<.001
	<20,000	13.58		11.15		14.41		24.02				
	20,000 to <35,000	9.21		7.96		9.60		14.86				
	35,000 to <50,000	13.10		11.61		13.91		17.42				
	50,000 to <75,000	18.79		17.43		20.43		16.41				
	≥75,000	45.33		51.85		41.66		27.29				
**Insurance**												.01
	No	7.42		6.16		8.93		5.27				
	Yes	92.58		93.84		91.07		94.73				
**Location**												.011
	Rural	12.26		10.64		13.55		14.10				
	Urban	87.74		89.36		86.45		85.90				

^a^CVD: cardiovascular disease.

^b^Low CVD risk: 0-1 risk factors.

^c^Moderate CVD risk: 2-3 risk factors.

^d^High CVD risk: 4-5 risk factors.

Among the included participants, 41.9% (weighted percentage) used mHealth to make health decisions, 50.8% (weighted percentage) used mHealth to track health progress toward a health-related goal, 18.3% (weighted percentage) used mHealth to share health information with health providers, and 37.7% (weighted percentage) used mHealth to discuss health decisions with health providers (Table 2). Significant differences were observed in the proportion of participants using mHealth to track health progress and share health information with health providers among the 3 CVD risk categories (all *P*s<.001).

**Table 2:** Weighted percentages and SEs of mHealth^a^ usage by cardiovascular risk among adults with or at risk for CVD^b^.

mHealth use	All, weighted percentage (SE)	Low CVD risk^c^, weighted percentage (SE)	Moderate CVD risk^d^, weighted percentage (SE)	High CVD risk^e^, weighted percentage (SE)	*P* value
Make health decisions	41.90 (0.9)	41.14 (1.5)	42.59 (1.3)	42.51 (3.1)	.76
Track health progress toward a health-related goal	50.78 (0.9)	53.81 (1.5)	49.53 (1.2)	38.02 (3.1)	<.001
Share health information with health providers	18.29 (0.6)	14.97 (0.9)	19.91 (0.9)	28.89 (1.9)	<.001
Discuss health decisions with health providers	37.72 (0.9)	34.94 (1.2)	38.10 (1.4)	40.52 (3.0)	.50

^a^mHealth, mobile health.

^b^CVD: cardiovascular disease.

^c^Low CVD risk: 0-1 risk factors.

^d^Moderate CVD risk: 2-3 risk factors.

^e^High CVD risk: 4-5 risk factors.

**Figure 2:**
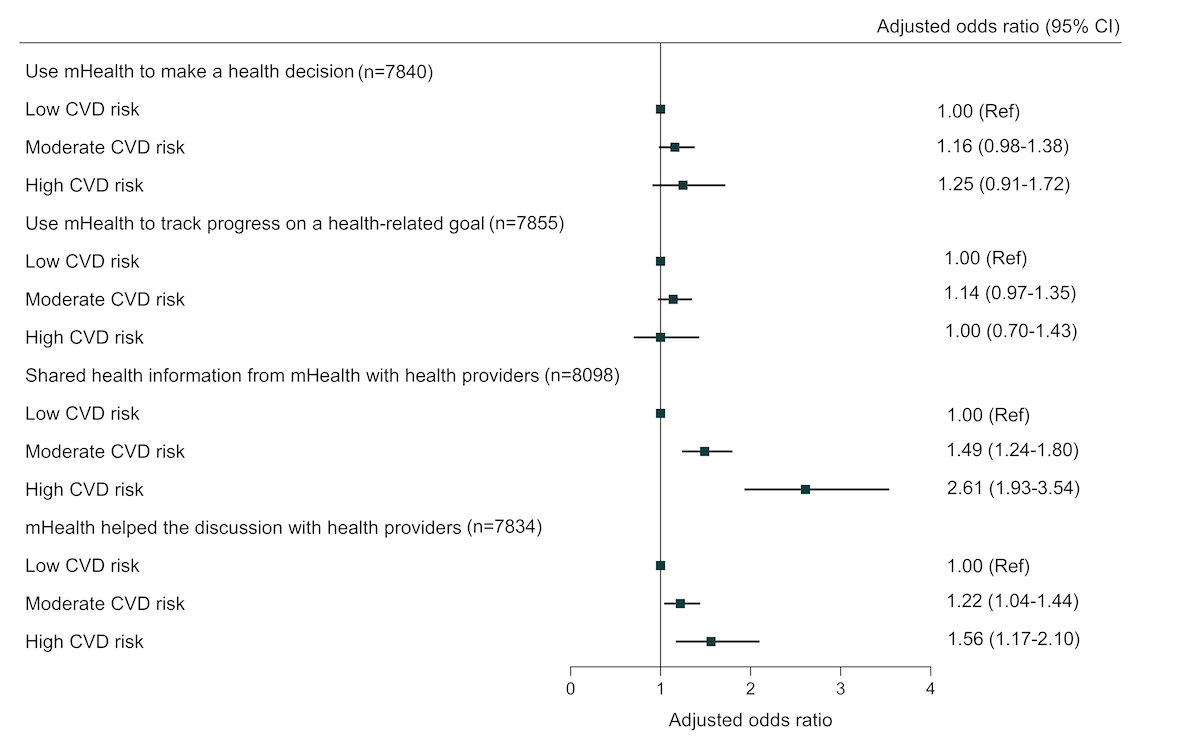
Associations between cardiovascular risk and mHealth use among adults with or at risk for CVD. The results are from weighted multivariable logistic regression models. Each logistic regression model was adjusted for age, sex, race, education, household income, location, and health insurance. Low CVD risk: 0-1 risk factors; moderate CVD risk: 2-3 risk factors; high CVD risk: 4-5 risk factors. CVD: cardiovascular disease; mHealth: mobile health; Ref: reference.

After adjusting for age, gender, race, education, household income, location, and health insurance, adults with moderate CVD risk were more likely to use mHealth to share health information with health providers (adjusted odds ratio 1.49, 95% CI 1.24-1.80) and to discuss health decisions with health providers (1.22, 95% CI 1.04-1.44) compared to individuals with low CVD risk (Figure 2). Adults with high CVD risk was associated with higher odds of using mHealth to share health information with health providers (2.61, 95% CI 1.93-3.54) and to discuss health decisions with health providers (1.56, 95% CI 1.17-2.10) when compared to those with low CVD risk (Figure 2). There were no significant associations found between moderate or high CVD risk and the use of mHealth for both making health decisions and tracking progress on health-related goals, as compared to adults with low CVD risk (Figure 2).

**Figure 3:**
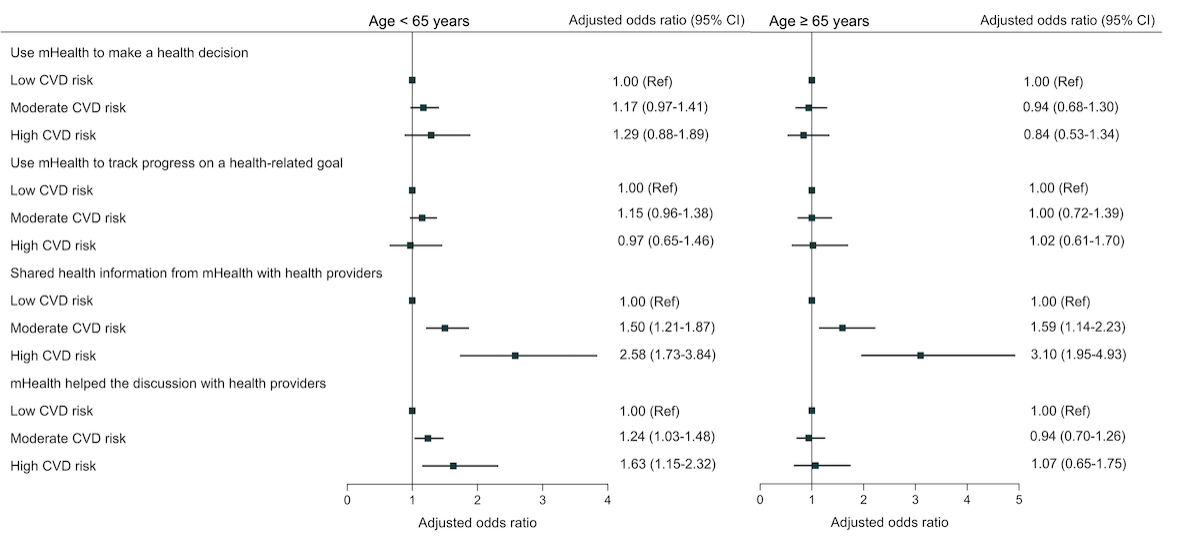
Associations between cardiovascular risk and mHealth use among older and younger adults with or at risk for CVD. Low CVD risk: 0-1 risk factors; moderate CVD risk: 2-3 risk factors; high CVD risk: 4-5 risk factors. Each weighted multivariable logistic regression model was adjusted for sex, race, education, household income, location, and health insurance. CVD: cardiovascular disease; mHealth: mobile health; Ref: reference.

Upon stratifying by age, we observed age disparities in the relationship between CVD risk and mHealth use (Figure 3). Among adults aged <65 years, adults with moderate or high CVD risk were more likely to use mHealth to share health information and discuss health decisions with health providers, compared to those with low CVD risk. Among adults aged ≥65 years, those with moderate CVD risk (1.59, 95% CI 1.14-2.23) and high CVD risk (3.10, 95% CI 1.95-4.93) were more inclined to use mHealth to share information with health providers, compared to individuals with low CVD risk. However, neither moderate nor high CVD risk were statistically significant factors associated with the use of mHealth for discussing health decisions with health providers in this age group.

**Figure 4:**
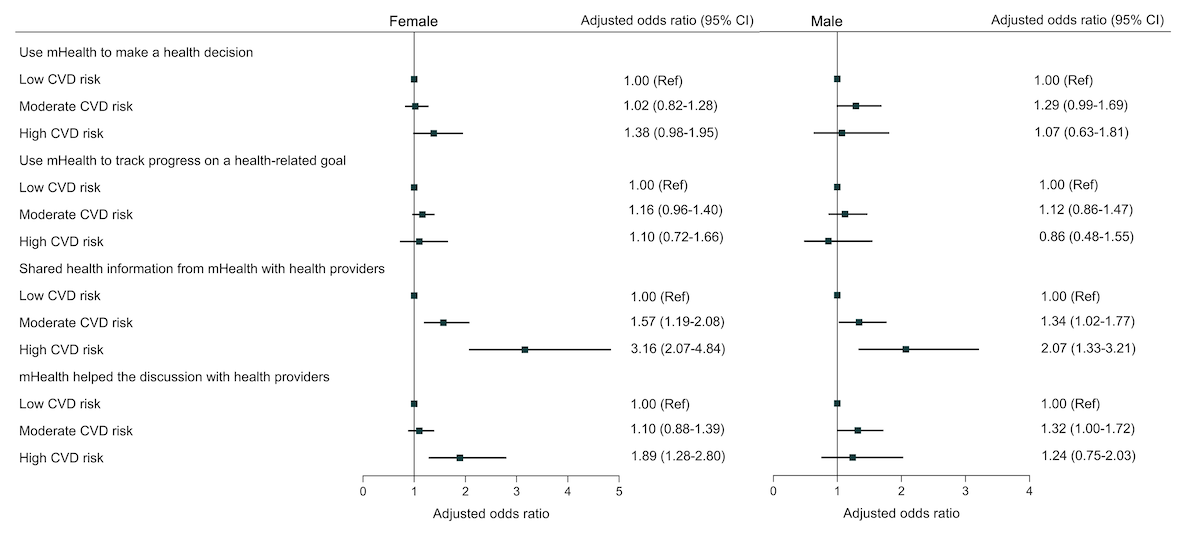
Associations between cardiovascular risk and mHealth use among women and men with or at risk for CVD. Low CVD risk: 0-1 risk factors; moderate CVD risk: 2-3 risk factors; high CVD risk: 4-5 risk factors. Each weighted multivariable logistic regression model was adjusted for age, race, education, household income, location, and health insurance. CVD: cardiovascular disease; mHealth: mobile health; Ref: reference.

After stratifying by gender, gender disparities in the odds of CVD risk associated with mHealth use were evident (Figure 4). For both women and men, having moderate and high CVD risk were significantly associated with increased odds of using mHealth to share health information with health providers when compared to adults with low CVD risk. Among women, having high CVD risk was associated with higher odds of using mHealth to discuss health decisions with health providers (1.89, 95% CI 1.28-2.80) compared with adults with low CVD risk; however, this significant association was not observed among men (1.24, 95% CI 0.75-2.03).

Our findings remained robust with the exclusion of individuals previously diagnosed as having heart disease (n=848) and with the exclusion of individuals aged ≥85 years (n=489; Table S3 in Multimedia Appendix 1).

In the assessment of the associations between each CVD risk factor and mHealth use (Table S4 in Multimedia Appendix 1), we observed that adults with diabetes (1.62, 95% CI 1.26-2.07), hypertension (1.88, 95% CI 1.54-2.30), and overweight or obesity (1.28, 95% CI 1.50-1.57) were more inclined to share health information through mHealth technology with health care providers compared to their counterparts. Additionally, adults who were overweight or obese (1.39, 95% CI 1.14-1.70) were more likely to use mHealth to track progress toward health-related goals than those with a normal BMI. Furthermore, adults with hypertension (1.38, 95% CI 1.19-1.61) were more inclined to use mHealth for discussions with health care providers compared to those without a hypertension diagnosis.

## Discussion

### Principal Findings

In this nationally representative sample, we observed that adults with moderate CVD risk (2-3 CVD risk factors) and high CVD risk (4-5 CVD risk factors) were more likely to use mHealth to share health information with health providers than those with low CVD risk (0-1 CVD risk factors). However, no significant associations were detected between moderate or high CVD risk and the usage of mHealth for both making health decisions and tracking progress toward health-related goals. These associations were consistent across various age groups and genders. Age and gender disparities in the likelihood of CVD risk being associated with the use of mHealth for discussing health decisions with health providers were evident. Specifically, among adults aged <65 years and women, individuals with high CVD risk were more inclined to use mHealth for discussing health decisions with health providers compared to those with low CVD risk.

### Comparison With Prior Work

Research has demonstrated that the usage of mHealth varies significantly by the presence of chronic conditions or CVD risk factors and demographics (including age, gender, race, educational levels, insurance, and region of residence) [10,15,17,19,24-26]. Numerous studies have explored disparities in mHealth use among individuals both with and without CVD risk factors [12,15]. These investigations have suggested that the relationship between CVD risk and mHealth use could potentially exhibit variations based on the total number of CVD risk factors present. To better explain the associations between CVD risk and mHealth use, we used multivariable logistic regression models, adjusting for multiple demographics identified in the previous studies [12,15]. To gauge the strength of the associations between CVD risk and mHealth use, we categorized individuals into 3 groups: low, moderate, and high CVD risk. These categories allowed us to assess the magnitude of the relationships between CVD risk and mHealth use effectively.

Our findings expand prior reports regarding mHealth use linked to CVD risk with additional patients and analyses. Shan and colleagues [12] used 2018 HINTS data to examine the association between mHealth use among individuals with or at risk for CVD. In their report using 2018 HINTS data [12], compared with adults with no history or risk factors for CVD, adults with CVD risk (defined as reporting a heart condition, diabetes mellitus, hypertension, or current smoking) were more likely to use mHealth to share health information with health providers [12]. In our study, which encompassed a larger and more extensive participant pool spanning 4 years, we likewise found that adults with moderate and high CVD risk (including type 2 diabetes mellitus, hypertension, current smoking, physical inactivity, and overweight or obesity) were more likely to use mHealth to share health information with their health providers compared to those with low CVD risk, corroborating the earlier research findings [12]. This trend suggests that adults with greater CVD risk may perceive mHealth as a valuable tool for enhancing communication with health care professionals, possibly due to a heightened awareness of their health status and the need for frequent monitoring and consultation [15]. This finding highlights the potential of mHealth to facilitate communication between patients and health care professionals, especially in populations at greater risk of CVD [12]. It is crucial to highlight that our study’s definition of CVD risk differs from the prior report. Unlike the previous study, our analysis includes factors such as physical inactivity and overweight or obesity but excludes heart conditions. Our study thoroughly examines the relationships between the composite CVD risk and mHealth use across various age groups and genders. To facilitate this study of the dose-response relationship, we categorized CVD risk into low, moderate, and high levels. The results revealed that adults with a moderate CVD risk (2-3 risk factors) were 1.5 times more likely and those with a high CVD risk (4-5 risk factors) were 2.6 times more likely to use mHealth for sharing health information with their health care providers, compared to those with a low CVD risk.

Furthermore, it is notable that we found adults with moderate and high CVD risk displayed a greater likelihood to use mHealth for engaging in discussions with health providers. This observation contradicts the results of a previous study, which reported no difference in the odds of using mHealth to facilitate discussions with health providers [12]. These discrepancies can potentially be attributed to the prior study’s exclusion of 2 crucial CVD risk factors: physical inactivity and obesity [12]. Additionally, that study did not investigate how mHealth use varies in relation to the various levels of multiple CVD risk factors [12]. However, our study revealed age and gender disparities in the likelihood of mHealth use for discussing health decisions with health providers. Specifically, among adults aged 65 years or younger, those with moderate or high CVD risk were more inclined to use mHealth for discussing health decisions with health providers compared to those with low CVD risk. These findings align with previous studies that older adults tend to be less likely to use the mHealth for making health-related decisions or discussions with health providers than younger adults [17,19,24,25]. This finding is not surprising, given that older adults may have lower levels of digital health literacy and be less capable of using smartphones, tablets, and complex health-related apps [32,33]. A prior study indicated that older adults might experience the first-level digital divide (lack of access to technologies) or the second-level digital divide (lack of use or skill in data input and analysis) [32]. Other barriers for older adults in mHealth use may include lack of knowledge in using mHealth, decreased sensory perception, poorly designed interface, absence of professional involvement, and the high cost of using mHealth [33,34]. With the rapidly increasing number of older adults globally [35], future researchers should address the barriers to mHealth use or access among older adults and develop person-centered mHealth-based interventions for CVD risk reduction [32].

In terms of gender, women with high CVD risk were more inclined to use mHealth for discussing health decisions with health providers compared to those with low CVD risk. These findings are similar to a previous study showing women with multiple chronic conditions had higher odds of using their mHealth technologies to discuss with their providers and manage their care [36]. The gender disparities may also reflect variations in health care–seeking behavior, with women tending to exhibit a stronger social motivation for engaging in health-related information searches, and they derive greater enjoyment from these activities [37]. These gender-specific digital inequalities suggest that gender differences should be acknowledged in developing mHealth interventions to empower CVD risk factors management [38].

While the primary focus of our study was to investigate the relationship between the composite CVD risk and mHealth use, we also explored the connection between various individual CVD risk factors and mHealth use. The findings revealed that adults with diabetes, hypertension, and overweight or obesity were more inclined to share health information using mHealth with health care providers. Moreover, overweight or obese individuals demonstrated a higher propensity to use mHealth for tracking health-related goals compared to those with a normal BMI. Additionally, adults with hypertension were more likely to engage in discussions with health care providers through mHealth compared to those without a hypertension diagnosis. These findings indicate that adults with multiple CVD health conditions, specifically diabetes and hypertension, might be more likely to use mHealth technology [12]. This raises the question of whether this pattern—higher CVD risk being associated with increased mHealth technology usage—varies based on the total number of CVD health conditions (including diabetes, hypertension, and hyperlipidemia) and CVD health-related behaviors (such as physical activity, smoking, and diet). Future research could expand its analyses by integrating additional CVD health conditions (eg, hyperlipidemia) and health-related behaviors (eg, diet) to attain a more comprehensive understanding of the relationship between the presence of CVD health conditions, CVD health behaviors, and mHealth use.

### Strengths and Limitations

This study used the recent 4 years of HINTS data, a nationally representative survey, to assess the associations between CVD risk and several specific mHealth use behaviors. There were several limitations in our study. First, this study was limited by the design nature (cross-sectional study design). Therefore, it is impossible to establish a cause-and-effect relationship between the composite CVD risk factor control and mHealth use. This study is also unable to assess the chronological relationship between the development of CVD risk factors and mHealth use. Second, there may be response bias due to the self-reported data. However, our analyses used the survey weighting method suggested by the HINTS statistical methodology. Third, hyperlipidemia is also a major CVD risk factor that is not included in this study due to the lack of such questions regarding hyperlipidemia in the 2017-2020 HINTS data set. Fourth, an important limitation of our study lies in the absence of adjustments for health conditions or illnesses, such as cancer, which could potentially impact the relationship between CVD risk and mHealth use. Future research should consider including health conditions or illnesses as covariates to further explore this association. Finally, a substantial proportion of missing data in the variables of interest raises concerns about potential bias in our findings. To mitigate this issue, future studies should employ suitable methods, such as multiple imputations, based on the underlying mechanisms causing the missing data.

### Conclusions

In this nationally representative sample of US adults regardless of whether they have a history of CVD or not, adults with a greater number of CVD risk factors displayed a heightened propensity to actively use mHealth technology for sharing health information and engaging in health-related discussions with their health providers. This trend was particularly pronounced among younger adults and women when compared to those with a low CVD risk. This suggests a promising avenue for improving health care communication and advancing both primary and secondary prevention efforts related to managing CVD risk factors through the effective usage of mHealth technology.

## Appendix

Multimedia Appendix 1 Unweighted frequencies and weighted multivariable logistic regression model results.

Multimedia Appendix 2 Original ChatGPT transcripts.

## Abbreviations

CVD: cardiovascular disease
HINTS: Health Information National Trends Survey
mHealth: mobile health
STROBE: Strengthening the Reporting of Observational Studies in Epidemiology
WHO: World Health Organization

## Data Availability

The data sets generated or analyzed during this study are available from the corresponding author on reasonable request.
