# Supplementary material for: Association Between the Composite Cardiovascular Risk and mHealth Use Among Adults in the 2017-2020 Health Information National Trends Survey: Cross-Sectional Study
Source: J Med Internet Res. 2024 Jan 4;26:e46277. doi: 10.2196/46277 (PMC10797506; doi:10.2196/46277)
Supplement: Multimedia Appendix 1 [file jmir_v26i1e46277_app1.docx]

**Supplemental material**

**Table S1.** Unweighted frequency of CVD risk factors among adults with or without CVD.

|  | **Without CVD**  **(n = 9725)** | **With CVD**  **(n = 806)** |
| --- | --- | --- |
| **Numbers of CVD risk factors** |  |  |
| 0 | 1261 | 31 |
| 1 | 2914 | 126 |
| 2 | 3032 | 228 |
| 3 | 1819 | 237 |
| 4 | 642 | 166 |
| 5 | 57 | 18 |
| **CVD risk** |  |  |
| Low CVD risk (0-1 risk factors) | 4175 | 157 |
| Moderate CVD risk (2-3 risk factors) | 4851 | 465 |
| High CVD risk (4-5 risk factors) | 699 | 184 |

CVD: cardiovascular disease.

**Table S2.** Unweighted frequency of demographic characteristics and mHealth usage by cardiovascular risk.

| **Characteristics** | | **All (N=10531)** | **Low CVD risk (n=4332)** | **Moderate CVD risk**  **(n=5316)** | **High CVD risk (n=883)** | ***P*** |
| --- | --- | --- | --- | --- | --- | --- |
| Age, Mean (SD) | | 54 (16.2) | 50 (16.9) | 57 (15.4) | 62 (11.8) | < .001 |
| Age | | | | | | < .001 |
|  | 18-34 years | 1520 | 960 | 543 | 17 |  |
|  | 35-49 years | 2208 | 1080 | 1020 | 108 |  |
|  | 50-64 years | 3386 | 1205 | 1815 | 366 |  |
|  | 65-74 years | 2202 | 703 | 1227 | 272 |  |
|  | 75+ years | 988 | 297 | 588 | 103 |  |
|  | Missing | 227 | 87 | 123 | 17 |  |
| Gender | | | | | | < .001 |
|  | Female | 5544 | 2417 | 2668 | 459 |  |
|  | Male | 4277 | 1650 | 2266 | 361 |  |
|  | Missing | 710 | 265 | 382 | 63 |  |
| Race/Ethnicity | | | | | | < .001 |
|  | Non-Hispanic White | 6217 | 2791 | 3010 | 416 |  |
|  | Non-Hispanic Black | 1256 | 321 | 754 | 181 |  |
|  | Hispanic | 1429 | 572 | 718 | 139 |  |
|  | Non-Hispanic Asian | 476 | 242 | 204 | 30 |  |
|  | Non-Hispanic Others | 356 | 148 | 180 | 28 |  |
|  | Missing | 797 | 258 | 450 | 89 |  |
| Education | | | | | | < .001 |
|  | Less than high school | 476 | 116 | 270 | 90 |  |
|  | High school graduate | 1483 | 470 | 819 | 194 |  |
|  | Some college | 2966 | 1022 | 1620 | 324 |  |
|  | Bachelor's degree | 5420 | 2648 | 2509 | 263 |  |
|  | Missing | 186 | 76 | 98 | 12 |  |
| Household income | | | | | | < .001 |
|  | < $20 000 | 1336 | 384 | 737 | 215 |  |
|  | $20 000-$35 000 | 1066 | 370 | 564 | 132 |  |
|  | $35 000-$50 000 | 1207 | 424 | 662 | 121 |  |
|  | $50 000-$75 000 | 1807 | 727 | 948 | 132 |  |
|  | ≥ $75 000 | 4196 | 2069 | 1924 | 203 |  |
|  | Missing | 919 | 358 | 481 | 80 |  |
| Insurance | | | | | | .739 |
|  | No | 474 | 199 | 240 | 35 |  |
|  | Yes | 9930 | 4087 | 5012 | 831 |  |
|  | Missing | 127 | 46 | 64 | 17 |  |
| Location | | | | | | .001 |
|  | Rural | 1145 | 412 | 629 | 104 |  |
|  | Urban | 9386 | 3920 | 4687 | 779 |  |
|  | Missing | 0 | 0 | 0 | 0 |  |
| **mHealth usage** | | | | | | |
| Make health decisions | |  |  |  |  | .537 |
|  | No | 5495 | 2327 | 2750 | 418 |  |
|  | Yes | 3847 | 1671 | 1881 | 295 |  |
|  | Missing | 1189 | 334 | 685 | 170 |  |
| Track health progress toward a health-related goal | |  |  |  |  | < .001 |
|  | No | 4943 | 1977 | 2528 | 438 |  |
|  | Yes | 4425 | 2033 | 2115 | 277 |  |
|  | Missing | 1163 | 322 | 673 | 168 |  |
| Share health information with health providers | |  |  |  |  | < .001 |
|  | No | 7806 | 3353 | 3880 | 573 |  |
|  | Yes | 2022 | 652 | 1100 | 270 |  |
|  | Missing | 703 | 327 | 336 | 40 |  |
| Discuss health decisions with health providers | |  |  |  |  | .193 |
|  | No | 5615 | 2416 | 2793 | 406 |  |
|  | Yes | 3722 | 1581 | 1834 | 307 |  |
|  | Missing | 1194 | 335 | 689 | 170 |  |

CVD: cardiovascular diseases.  mHealth: mobile health. SD: standard deviation.

Low CVD risk: 0-1 risk factors; Moderate CVD risk: 2-3 risk factors; High CVD risk: 4-5 risk factors

**Table S3.** Associations between cardiovascular risk factors and mHealth usage (sensitivity analyses).^‡^

|  | **aOR (95% CI) ^a^** | **aOR (95% CI) ^b^** |
| --- | --- | --- |
| Used smartphone/tablet to make a health decision |  |  |
| Low CVD risk | 1.00 (ref) | 1.00 (ref) |
| Moderate CVD risk | 1.19 (0.99-1.44) | 1.16 (0.98-1.39) |
| High CVD risk | 1.23 (0.87-1.75) | 1.27 (0.92-1.75) |
| Used smartphone/tablet to track progress on a health-related goal |  |  |
| Low CVD risk | 1.00 (ref) | 1.00 (ref) |
| Moderate CVD risk | 1.12 (0.96-1.33) | 1.15 (0.97-1.36) |
| High CVD risk | 0.92 (0.64-1.33) | 0.99 (0.69-1.43) |
| Shared health information from a smartphone/tablet with health providers |  |  |
| Low CVD risk | 1.00 (ref) | 1.00 (ref) |
| Moderate CVD risk | **1.47 (1.21-1.79)** | **1.50 (1.24-1.81)** |
| High CVD risk | **2.55 (1.81-3.58)** | **2.60 (1.90-3.55)** |
| Smartphones/tablets helped the discussion with health providers |  |  |
| Low CVD risk | 1.00 (ref) | 1.00 (ref) |
| Moderate CVD risk | **1.21 (1.01-1.44)** | **1.22 (1.03-1.43)** |
| High CVD risk | **1.42 (1.05-1.91)** | **1.54 (1.14-2.07)** |

^‡^Results from weighted multivariable logistic regression models. Reference group: low CVD risk. Each logistic regression model was adjusted for age, sex, race, education, household income, location, and health insurance.

**^a^** Exclusion of individuals with heart disease (n = 806).

**^b^** Exclusion of individuals aged ≥ 85 years (n = 204).

CV: cardiovascular; mHealth: mobile health; *OR*: odds ratio; CI*:* confidence interval.

Low CVD risk: 0-1 risk factors; Moderate CVD risk: 2-3 risk factors; High CVD risk: 4-5 risk factors.

**Bold**: statistically significant.

**Table S4.** Associations between each cardiovascular risk factor and each component of mHealth usage.

|  | | **Weighted aOR (95% CI)** | | | |
| --- | --- | --- | --- | --- | --- |
| **Cardiovascular disease risk factors** | | **Model 1: Used smartphone/tablet to make a health decision**  **(n = 7840)** | **Model 2: Used smartphone/tablet to track progress on a health-related goal**  **(n = 7855)** | **Model 3: Shared health information from a smartphone/tablet with health providers**  **(n = 8098)** | **Model 4: Smartphones/tablets helped the discussion with health providers**  **(n = 7834)** |
| **Diabetes** |  |  |  |  |  |
|  | No | 1.00 (ref) | 1.00 (ref) | 1.00 (ref) | 1.00 (ref) |
|  | Yes | 1.08 (0.85-1.36) | 1.14 (0.88-1.47) | **1.62 (1.26-2.07)** | 1.08 (0.86-1.36) |
| **Hypertension** |  |  |  |  |  |
|  | No | 1.00 (ref) | 1.00 (ref) | 1.00 (ref) | 1.00 (ref) |
|  | Yes | 1.03 (0.84-1.26) | 1.12 (0.90-1.39) | **1.88 (1.54-2.30)** | **1.38 (1.19-1.61)** |
| **Current Smoking** |  |  |  |  |  |
|  | No | 1.00 (ref) | 1.00 (ref) | 1.00 (ref) | 1.00 (ref) |
|  | Yes | 1.11 (0.85-1.45) | 0.74 (0.54-1.01) | 0.91 (0.61-1.34) | 1.18 (0.88-1.60) |
| **Overweight/obese** |  |  |  |  |  |
|  | No | 1.00 (ref) | 1.00 (ref) | 1.00 (ref) | 1.00 (ref) |
|  | Yes | 1.21 (0.99-1.48) | **1.39 (1.14-1.70)** | **1.28 (1.50-1.57)** | 1.14 (0.97-1.34) |
| **Unregular PA** |  |  |  |  |  |
|  | No | 1.00 (ref) | 1.00 (ref) | 1.00 (ref) | 1.00 (ref) |
|  | Yes | 0.94 (0.78-1.12) | 0.84 (0.71-1.00) | 0.93 (0.79-1.09) | 0.91 (0.77-1.08) |

^‡^Results from weighted multivariable logistic regression models, adjusting for age, sex, race, education, household income, location, and health insurance.

aOR: adjusted odds ratio; CI*:* confidence interval; PA: physical activity.

**Bold**: statistically significant.
