# Supplementary material for: Association Between the Composite Cardiovascular Risk and mHealth Use Among Adults in the 2017-2020 Health Information National Trends Survey: Cross-Sectional Study
Source: J Med Internet Res. 2024 Jan 4;26:e46277. doi: 10.2196/46277 (PMC10797506; doi:10.2196/46277)
Supplement: Multimedia Appendix 2 [file jmir_v26i1e46277_app2.docx]

**Original ChatGPT Transcripts**

Default (GPT-3.5) script

revise: the prevalence of uncontrolled CVD risk factors remains high among adults with or without CVD

ChatGPT

The high prevalence of uncontrolled cardiovascular disease (CVD) risk factors persists among both adults with and without a history of CVD.

revise: No significant associaitons were found in relation to the CVD risk and the use of mHealth for both making a health decision and tracking progress on a health-related goal.

ChatGPT

No significant associations were identified regarding the use of mHealth for making health decisions and tracking progress on health-related goals in relation to cardiovascular disease (CVD) risk.

No significant associaitons were found between moderate or high CVD risk and the use of mHealth for both making a health decision and tracking progress on a health-related goal, compared to low CVD risk

ChatGPT

There were no significant associations found between moderate or high cardiovascular disease (CVD) risk and the use of mHealth for making health decisions and tracking progress on health-related goals, as compared to individuals with low CVD risk.

Among adults aged ≥ 65 years, adults with moderate CVD risk ([1.59 [1.14-2.23]] and high CVD risk (3.10 [1.95-4.93]) were more likely to use mHealth to share information with health providers, compared to individuals with low CVD risk; however, this associations were not significant for using mHealth to discuss health decisions with health providers among this age group.

ChatGPT

Among adults aged 65 years and older, those with moderate cardiovascular disease (CVD) risk (odds ratio: 1.59 [95% confidence interval: 1.14-2.23]) and high CVD risk (odds ratio: 3.10 [95% confidence interval: 1.95-4.93]) were more inclined to use mHealth for sharing information with healthcare providers when compared to individuals with low CVD risk. However, it's worth noting that these associations were not statistically significant for using mHealth to discuss health decisions with healthcare providers in this age group.

However, both moderate and high CVD risk were not statistically significant for using mHealth to discuss health decisions with healthcare providers in this age group.

ChatGPT

However, it's important to note that neither moderate nor high CVD risk were statistically significant factors associated with the use of mHealth for discussing health decisions with healthcare providers in this age group.

There were no significant associations found between moderate or high CVD risk and the use of mHealth for both making health decisions and tracking progress on health-related goals, as compared to adults with low CVD risk

ChatGPT

No significant associations were observed between moderate or high cardiovascular disease (CVD) risk and the utilization of mHealth for both making health decisions and monitoring progress on health-related goals when compared to adults with low CVD risk.

summarize: Upon stratifying by age, we observed age disparities in the relationship between CVD risk and mHealth usage (Figure 2). Among adults aged < 65 years, adults with moderate or high CVD risk were more likely to use mHealth to share health information and discuss health decisions with health providers, compared to those with low CVD risk. Among adults aged ≥ 65 years, those with moderate CVD risk ([1.59 [1.14-2.23]] and high CVD risk (3.10 [1.95-4.93]) were more inclined to use mHealth to share information with health providers, compared to individuals with low CVD risk. However, neither moderate nor high CVD risk were statistically significant factors associated with the use of mHealth for discussing health decisions with health providers in this age group. After stratifying by gender, gender disparities in the odds of CVD risk associated with mHealth usage were evident (Figure 3). For both females and males, having moderate and high CVD risk were significantly associated with increased odds of using mHealth to share health information with health providers when compared to adults with low CVD risk. Among females, having high CVD risk was associated with higher odds of using mHealth to discuss health decisions with health providers (1.89 [1.28-2.80]) compared with adults with low CVD risk; however, this significant association was not observed among males (1.24 [0.75-2.03]).

ChatGPT

In the study, age and gender disparities were observed in the relationship between cardiovascular disease (CVD) risk and the use of mHealth (Figure 2 and Figure 3). Among adults under 65 years old, those with moderate or high CVD risk were more likely to use mHealth for sharing health information and discussing health decisions with healthcare providers compared to those with low CVD risk. Among adults aged 65 and older, individuals with moderate CVD risk (odds ratio: 1.59 [95% CI: 1.14-2.23]) and high CVD risk (odds ratio: 3.10 [95% CI: 1.95-4.93]) were more inclined to use mHealth to share information with health providers, but neither moderate nor high CVD risk were statistically significant factors associated with using mHealth to discuss health decisions in this age group.

When stratified by gender, gender disparities were evident. Both females and males with moderate and high CVD risk had significantly higher odds of using mHealth to share health information with healthcare providers compared to adults with low CVD risk. Among females, having high CVD risk was also associated with higher odds of using mHealth to discuss health decisions with healthcare providers (odds ratio: 1.89 [95% CI: 1.28-2.80]) compared to those with low CVD risk. However, this significant association was not observed among males (odds ratio: 1.24 [95% CI: 0.75-2.03]).

revise: There were no significant associations found between moderate or high CVD risk and the use of mHealth for both making health decisions and tracking progress on health-related goals, as compared to adults with low CVD risk

ChatGPT

No significant associations were detected between moderate or high cardiovascular disease (CVD) risk and the utilization of mHealth for making health decisions and monitoring progress on health-related goals when compared to adults with low CVD risk.

summarize:adults with moderate CVD risk were more likely to use mHealth to share health information with health providers (aOR: 1.49, 95% CI: 1.24-1.80) and to discuss health decisions with health providers (1.22 [1.04-1.44]) compared to individuals with low CVD risk (Figure 1). Adults with high CVD risk was associated with higher odds of using mHealth to share health information with health providers (2.61 [1.93-3.54]) and to discuss health decisions with health providers (1.56 [1.17-2.10]) when compared to those with low CVD risk (Figure 1).

ChatGPT

In the study, adults with moderate cardiovascular disease (CVD) risk had a higher likelihood of using mHealth to share health information with healthcare providers (adjusted odds ratio [aOR]: 1.49, 95% confidence interval [CI]: 1.24-1.80) and to discuss health decisions with healthcare providers (aOR: 1.22, 95% CI: 1.04-1.44) when compared to individuals with low CVD risk (Figure 1). Moreover, adults with high CVD risk were also associated with increased odds of using mHealth for sharing health information with healthcare providers (aOR: 2.61, 95% CI: 1.93-3.54) and for discussing health decisions with healthcare providers (aOR: 1.56, 95% CI: 1.17-2.10) when compared to those with low CVD risk (Figure 1).

The higher CVD risk, the higher odds of using mhealth to share health information with health providers

ChatGPT

Indeed, there is a positive correlation between higher cardiovascular disease (CVD) risk and an increased likelihood of using mHealth to share health information with healthcare providers.

The association between CVD risk and the use of mHealth for making health decisions with health providers are differed by age and gender

ChatGPT

The relationship between cardiovascular disease (CVD) risk and the use of mHealth for making health decisions with healthcare providers varies depending on age and gender.

Age and gender disparities in the odds of CVD risk associated with the use of mHealth to make discuss health decisions with health providers were evident

ChatGPT

Age and gender disparities in the likelihood of cardiovascular disease (CVD) risk being associated with the use of mHealth for discussing health decisions with healthcare providers were observed.
